# Thromboembolic Risk and High Prothrombotic Factors in Childhood Acute Lymphoblastic Leukemia with Ischemic Stroke: A Literature Review of Personalized and Institutional Approaches to Prophylaxis

**DOI:** 10.3390/jpm15060228

**Published:** 2025-06-02

**Authors:** Marta Malczewska, Ewa Dudkiewicz, Joanna Zawitkowska, Monika Lejman

**Affiliations:** 1 Department of Pediatric Hematology, Oncology, and Transplantology, University Hospital for Children, Antoniego Gębali 6, 20-093 Lublin, Poland; martmalczewska@gmail.com (M.M.); ewa.dudkiewicz@uszd.lublin.pl (E.D.); 2 Department of Pediatric Hematology, Oncology, and Transplantology, Medical University of Lublin, Antoniego Gębali 6, 20-093 Lublin, Poland; 3 Independent Laboratory of Genetic Diagnostics, Medical University of Lublin, Antoniego Gębali 6, 20-093 Lublin, Poland; monika.lejman@umlub.pl

**Keywords:** acute lymphoblastic leukemia, ischemic stroke, prothrombic factors, antithrombotic prophylaxis, thromboembolic risk, low-molecular-weight heparin

## Abstract

**Background**: Although thromboembolic complications are recognized in the treatment of acute lymphoblastic leukemia (ALL), ischemic strokes are rare but severe events. These life-threatening complications not only pose an immediate risk but can also result in long-term neurological deficits, significantly impacting a patient’s quality of life. Identifying high-risk patients and implementing effective prophylaxis strategies are crucial for improving patient outcomes. In addition to strokes, these patients are also at risk of other embolic and thrombotic events, which can occur in up to 35% of patients. Despite this, there are still no clear guidelines for prophylactic management in pediatric patients treated for oncologic diseases. **Results**: Using the example of a 14-year-old male treated for ALL who suffered an ischemic stroke, we conducted a review of the literature on embolic and thrombotic events, neurological complications, methods of prevention, and ways to monitor and detect patients with an increased risk of such difficulties. We outlined our approach to the monitoring of prothrombotic factors, the interpretation of their levels, and the subsequent adjustment to prophylactic management based on these findings. As a result of this review, we reached two basic conclusions. First, thromboembolic episodes are not uncommon complications in pediatric patients and can cause long-lasting consequences, even after the cancer is cured. Secondly, despite such an urgent problem, clinicians are still struggling with the question of monitoring prothrombotic factors, the choice of drug, and the duration of prophylaxis. Their decisions depend on the experience of the treating center. **Conclusions:** The pediatric population being treated for malignant disease urgently requires the establishment of guidelines that standardize the management of thromboembolic events.

## 1. Introduction

Complications associated with the treatment of acute lymphoblastic leukemia (ALL) have become a challenge for modern medicine as they effectively limit the use of conventional chemotherapy and also are the cause of decreased survival rates. Acute treatment complications occur in nearly 95% of patients [1]. A desire to prevent complications contributes firstly to the development of new methods for their prevention and treatment, and secondly to new methods of treating cancer itself. Treatment complications encompass a spectrum of issues, ranging from bone marrow aplasia, infections, and metabolic disorders (such as diabetes, hypertriglyceridemia, hypoalbuminemia, and electrolyte imbalances) to acute damage of the liver, kidneys, and pancreas, along with the nervous system. Additionally, patients can experience clotting disorders, which can include both bleeding and thromboembolic episodes [2]. Coagulation abnormalities in patients undergoing treatment for acute lymphoblastic leukemia can result from cancer as well as from the drugs used, particularly steroids and asparaginase (ASP); additional factors include the presence of a central venous catheter, infection, immobilization, and obesity [3,4]. To monitor and predict the occurrence of thromboembolic episodes, various parameters are evaluated in patients. These include basic clotting times, protein C and protein S levels, antithrombin III levels, fibrinogen levels, D-dimers, factor V Leiden, factor VIII, and von Willebrand factor activity and antigen. Depending on the clinical situation, imaging studies may be performed. Elevated prothrombotic factors significantly increase the risk of a thromboembolic episode. Thus, the active monitoring of these factors allows an appropriate administration of prophylaxis. We believe that monitoring prothrombotic factors during steroid therapy and the administration of subcutaneous heparin prophylactically is safe and effective in decreasing the risk of thrombotic episodes. In addition to monitoring these factors, it is essential to assess clotting times and platelet counts. Therefore, we conducted a comprehensive literature review of thromboembolic complications in pediatric ALL, with a particular focus on risk factors, pathophysiology, and preventive strategies. To illustrate the real-life challenges of thromboembolism monitoring and management, we refer to the clinical course of a 14-year-old patient treated for ALL. In our center, we have established a personalized approach to thromboprophylaxis based on monitoring key prothrombotic factors, including factor VIII and von Willebrand factor activity and antigen. Depending on their dynamic changes, patients receive low-molecular-weight heparin prophylaxis. Supplementation with antithrombin III and fibrinogen is introduced as needed. This individualized management strategy was applied in the case of the male patient described in this study.

## 2. Materials and Methods

A targeted literature review was conducted to identify relevant studies on thromboembolic complications in pediatric patients with acute lymphoblastic leukemia. The databases PubMed, Scopus, and Google Scholar were systematically searched using a combination of the following keywords: “ischemic stroke”, “thromboembolic complications”, “acute lymphoblastic leukemia”, “pediatric oncology”, “neurological complications”, and “stroke prevention in children”. The search included articles published between 2005 and 2024, with a restriction to English-language full texts. Eligible publications included case reports, original research articles, systematic reviews, and meta-analyses. Studies focusing exclusively on adult populations, those lacking clinical relevance, or containing purely theoretical content without application to patient care were excluded. The identified literature was assessed for data on incidence, risk factors, preventive strategies, and challenges in the clinical management of thromboembolic events in children with ALL. Particular attention was given to publications proposing individualized or risk-adapted approaches to thromboprophylaxis, in line with the personalized medicine perspective. For the case presentation, clinical data were obtained from a retrospective review of the patient’s electronic medical records, including physical examination findings, patient history, laboratory test results, and neuroimaging studies available from the hospital information system. Written informed consent for the use of anonymized medical data was obtained from the patient’s legal guardian.

## 3. Case Presentation

A fourteen-year-old male was admitted to our hospital due to suspected cancer. The medical history included subfebrile states, weakness, fatigue, and blood count abnormalities that had persisted for more than two weeks before hospitalization. The family history was irrelevant. At the time of admission, he was in good condition. The physical examination revealed pale skin, petechiae, bruising, tachycardia, and an enlarged spleen. His BMI (Body Mass Index) was 21.67, which was normal. A series of laboratory and imaging studies were performed. The laboratory results are presented in Table 1; abnormal results are in bold. In the morphology, we observed hyperleukocytosis, anemia, thrombocytopenia, and the presence of undifferentiated cells. The LDH (lactate dehydrogenase) level was elevated (the laboratory standard at our hospital is less than 350). Protein S levels were initially low but retesting 10 days later showed normal levels, leading to the discontinuation of a further diagnosis. Additionally, C-reactive protein and the von Willebrand antigen showed a slight elevation. The abnormality found during abdominal ultrasound was a massive spleen measuring 261 mm × 82 mm.

The patient was diagnosed with pre-B acute lymphoblastic leukemia and started treatment according to the AIEOP-BFM 2017 Protocol in July 2022. Genetic analyses conducted on the bone marrow samples included basic genetic tests to investigate known rearrangements of *BCR::ABL1* (9,22), *KMT2A* (11q23), *ETV6::RUNX1* (12;21), and *TCF3* (19p13.3) using the FISH (fluorescent in situ hybridization) method. Additionally, chromosome banding analyses were performed on bone marrow samples using standard methods. The patient presented the following abnormal, unbalanced, and complex somatic karyotype: mos 44~45,XY,del(3)(q13),der(7)t(7;17)(p12;q12),der(9)t(3;9)(q21;p13),dic(9;12)(p13;p11),+mar((16))/46,XY((4)).ish der(7)t(7;17)(p12;q12)(wcp7+;wcp17+)((3)),der(9)t(3;9)(q21;p13)(wcp3+;wcp9+),dic(9;12)(p13;p11)(wcp9+)((3)). The patient was classified for an analysis of the *IKZF1*plus status. A DNA microarray test confirmed the deletion of the *IKZF1*, *CDKN2A*, *CDKN2B*, and *PAX5* genes. Based on the results, the patient met the criteria for *IKZF1*plus. RNA sequencing and FISH testing excluded the activation of the JAK-STAT signaling pathway genes *JAK2*, *CRLF2*, *IL7R*, *EP0R*, and *IGH* or the activation of ABL family genes *ABL1*, *ABL2*, *PDGFRB*, and *CSF1R*. Due to a positive response to treatment, the child was classified into the standard risk group. The patient continued treatment with Protocol M and Protocol II and, throughout this course, no serious complications were observed. Table 2 shows the cytostatic used in each phase of the protocol.

**Figure 1:**
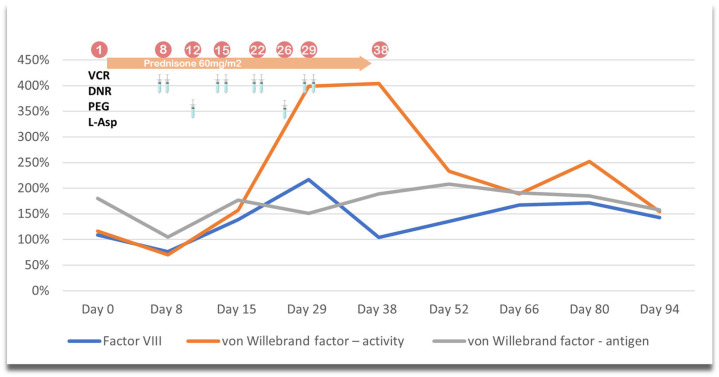
Graphical representation of changes in prothrombotic factors during Protocol I.

**Figure 2:**
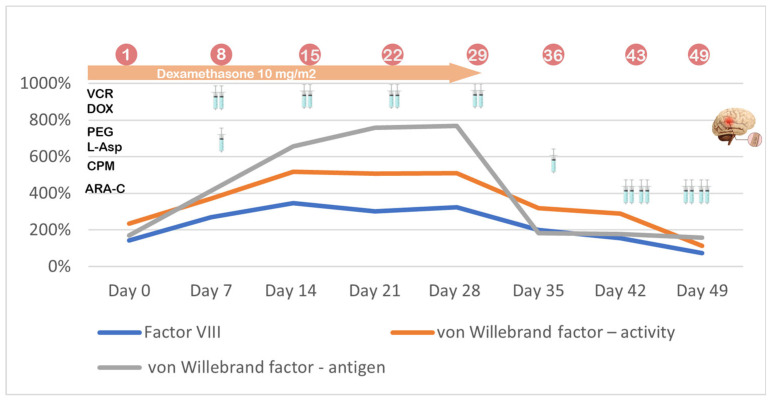
Graphical representation of changes in prothrombotic factors during Protocol II.

Throughout treatment, the patient’s coagulation parameters, including clotting times, antithrombin III (AT-III), and fibrinogen levels, were carefully monitored. The frequency of measurements was adjusted based on the child’s clinical condition, typically twice a week. As part of assessing the risk of thromboembolic events, three key prothrombotic factors were also tracked. During Protocol I, monitoring focused on two critical time points—days 8 and 15—during which the response to treatment was assessed. Additional assessments were conducted at the start and the end of steroid reduction. Following these key milestones, measurements were taken every two weeks until the prothrombotic factors normalized. In Protocol II, the prothrombotic parameters were monitored weekly, providing a consistent overview of the patient’s progress. The reference ranges for these factors vary based on blood type. Our patient’s blood type was AB, Rh D positive. For this group, the norms are as follows: factor VIII (50–150%), von Willebrand factor (60.8–239.8%), and von Willebrand antigen (66.1–176.2%). The laboratory standards for antithrombin in our hospital range from 83% to 128%, while for fibrinogen, the range is 2.00 to 4.00 g/L. In cases of reduced antithrombin III or fibrinogen levels, the patient is administered lyophilized antithrombin or cryoprecipitate in appropriate doses. The dynamics of the selected prothrombotic factors as well as antithrombin III and fibrinogen levels during Protocol II are presented in Table 3. Figure 1 and Figure 2 graphically illustrate the differences in the analyzed factors compared with the received treatment during Protocols I and II. Values above the norm are shown in bold, while those below the norm are underlined.

An analysis of the data revealed that prothrombotic factors were much higher during Protocol II than in Protocol I. In contrast, normalization of these factors occurred within a few days after the end of steroid therapy during Protocol II. Conversely, during Protocol I, elevated factors were observed a couple of weeks after steroid withdrawal. During Protocol I, von Willebrand factor activity reached the highest values, while during Protocol II, the highest values were obtained by von Willebrand antigen. In light of the above details, we decided to include a prophylactic dose of 1 mg/kg body weight of low-molecular-weight heparin during the patient’s treatment.

Two days after completing the intensive part of treatment—Protocol II—the patient experienced a tonic-clonic seizure, complicated by sudden cardiac arrest. At that point, the patient had not received low-molecular-weight heparin for two days. It had been discontinued following the completion of the intensive phase of treatment and because of low levels of prothrombotic factors. Additionally, the patient was in a phase of bone marrow aplasia after chemotherapy, with a platelet count below 50,000/µL. After successful resuscitation, restoration of spontaneous circulation was achieved. The patient was then transferred to the Intensive Care Unit. His condition remained stable but required ventilator therapy. At the time of the incident, laboratory analyses were conducted, with the results presented in Table 4. According to the relevant findings, pancytopenia was observed in the morphology (after chemotherapy was administered). The levels of AspAT, C-reactive protein, fibrinogen, and D-dimers were elevated. Abdominal ultrasonography did not show any abnormalities, including abnormal hepatic vascular flow.

**Figure 3:**
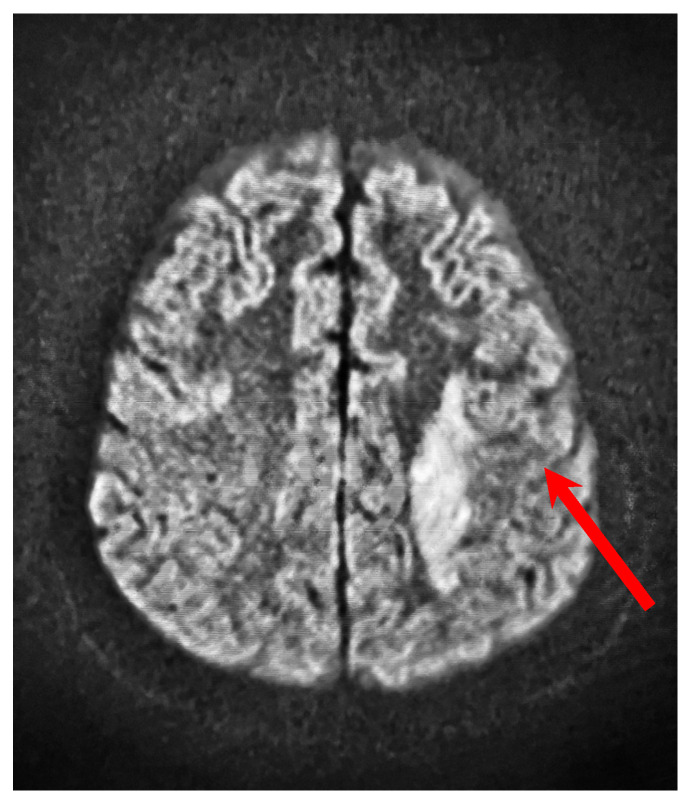
MRI scan of the head—baseline. The red arrow indicates the stroke focus.

MRI of the head showed a focus of an ischemic stroke on the left side, with a diameter of 62 × 20 mm. Figure 3 shows the stroke focus in the MRI scan of the head.

After 2 days the patient was extubated; his condition was stable, so he was transferred back to the Pediatric Hematology, Oncology and Transplantology Unit. A physical examination revealed muscle weakness on the right side, deviation of the tongue and palatal uvula, an asymmetrical smile, and positive Babinski signs on the right side. As an extension of the thrombophilia diagnosis, we assessed anti-β2-glycoprotein antibodies, anti-cardiolipin antibodies, and lupus anticoagulant, all of which returned negative. DNA sequencing was performed to analyze mutations in the *F2* (Coagulation Factor II), *F5* (Coagulation Factor V), *MTHFR*, and *SERPINE1* genes using the Sanger method to rule out congenital thrombophilia. The analysis targeted pathogenic variants, including p.Arg534Gln in the *F5* gene, variant 20210G>A in the *F2* gene, and variants p.Ala222Val and p.Glu429Ala in the *MTHFR* gene. None of these mutations were detected in the specified genes. Additionally, a guanine nucleotide insertion/deletion polymorphism (4G/5G variant) within the promoter of the *PAI* gene (*SERPINE1*) was examined, revealing a heterozygous pattern in one allele. This identified genotype can correlate with an elevated risk of thrombophilia, particularly when combined with other predisposing factors. For a month, the patient underwent intensive rehabilitation in the Department. His condition also allowed the initiation of maintenance treatment. The hemiparesis completely resolved; regular follow-up MRIs indicated a progressive reduction in the size of the ischemic focus. Currently, he is alive and well.

## 4. Literature Review

Pediatric ALL is associated with the highest risk of thrombosis compared with other childhood cancers. The incidence of thrombosis ranges from 1% to 36% [5]. According to the literature, the mortality rate in the pediatric population is approximately 2.2%. However, when cerebral venous sinus thrombosis is involved, the mortality rises to 6.25% [6]. The variation in reported thromboembolic event (TE) incidence across the literature can be attributed to differences in study designs, the prospective or retrospective nature of the studies, the inclusion of asymptomatic cases, and the specific protocols used at each medical center. For example, the BFM-90 study reported a 1.7% incidence of thrombosis among 1100 treated children. In contrast, Korte et al. observed an incidence of 14.3%, which included patients treated for both acute lymphoblastic leukemia (ALL) and non-Hodgkin lymphoma (NHL). The Japan Association of Childhood Leukemia Study reported two cases of hemostatic disorders among 127 patients, accounting for 1.6%. One of these patients suffered an ischemic stroke [5]. In a 2023 study by Guzelkucuk et al. involving 3968 patients, 70 embolic complications were identified, with 1.8% involving the central nervous system (CNS). It is noteworthy that in five patients (7%) diagnosed with cerebral vein thrombosis, persistent neurological sequelae such as epilepsy and neurological deficits were observed [7]. A 2022 study involving 652 patients undergoing treatment for acute lymphoblastic leukemia documented embolic episodes in 8.7% of cases [8].

Thromboembolic events most commonly affect children under the age of one, with a secondary peak incidence observed in adolescents aged 11 to 18. Nearly all cases occur in hospitalized children, with only 5% classified as ‘unprovoked’. Central venous catheters (CVCs) are major contributors, causing thrombosis in an estimated 90% of newborns and nearly 50% of adolescents. Due to their use, most cases involve the upper central venous system [5]. Other risk factors can also include a family history of thrombosis, the presence of a mediastinal mass, belonging to a high-risk treatment group, congenital thrombophilia, severe infections, malignancies, congenital heart defects, inflammatory bowel disease, nephrotic syndrome, recent surgery, musculoskeletal trauma, prolonged immobilization, and obesity [9]. Nevertheless, the malignancy itself—together with the therapeutic agents used in its treatment—remains the most significant prothrombotic issue. Cancer cells contribute to thrombosis by activating coagulation factors (VIII, IX, vWF, and alpha-2-macroglobulin), secreting cytokines, and inducing endothelial damage [10,11]. Given the diversity of risk factors, ranging from treatment-related exposures to underlying patient characteristics, individual risk profiling becomes an essential component of preventive care. Thromboembolic events typically occur during the induction phase of treatment, in which systemic steroids and asparaginase constitute the core components of the protocol [12]. Systemic steroids enhance the production of prothrombotic factors, including factors XII, XI, IX, X, VIII, VII, V, and II. Asparaginase, in contrast, reduces the synthesis of both procoagulant and anticoagulant proteins such as fibrinogen, plasminogen, and antithrombin III [13,14]. Their combined use also leads to elevated levels of von Willebrand factor antigen and its high-molecular-weight multimers [15]. Consequently, normal anticoagulant function is impaired, disrupting thrombin regulation and promoting a prothrombotic state. These pathophysiological mechanisms support the rationale for close laboratory surveillance during therapy, enabling risk-adapted modifications to anticoagulation management.

The Padua Prediction Score is a well-established tool for evaluating venous thromboembolism (VTE) risk in hospitalized adults and is commonly used in everyday clinical practice. According to the Padua score, the risk factors for VTE include active cancer (3 points), immobilization (3 points), a history of previous venous thromboembolism (3 points), congenital thrombophilia (3 points), recent surgery or trauma (within the last month) (2 points), age over 70 years (1 point), heart or respiratory failure (1 point), recent myocardial infarction or ischemic stroke (1 point), acute infection (1 point), obesity (1 point), and hormonal therapy (1 point). A score of 4 or more points indicates a significant risk of thrombosis [16]. In pediatric patients, particularly in the early stages of treatment, the score is typically between 5 and 8 points. Although the Padua scale is not directly applied to children, it remains a helpful tool for clinicians considering the overlap in risk factors between adults and pediatric populations.

Thromboembolic events can affect treatment outcomes. A 2021 report from the Cancer in Young People anada (CYP-C) group highlighted that patients who experienced thrombosis exhibited lower survival rates. A retrospective analysis of children treated for ALL revealed that those who had a thrombotic episode showed significantly lower overall survival (OS) rates (68.8% vs. 87.2%) and event-free survival (EFS) rates (80.2% vs. 93.7%) [17]. The Dutch Childhood Oncology Group (DCOG) ALL-10 (2004–2013) treated 778 children aged 1–18 years who had ALL (T or B cell), including 59 reported cases of VTE (7.6%), 26 of which were cerebral venous sinus thrombosis (CSVT) (44.1%). Four patients with CSVT died, one due to a CNS event and intracranial hemorrhage. Neurological morbidity was reported in five cases [18]. Neurological complications can represent important clinical manifestations of thromboembolic disease and serve as critical indicators of its presence. Notably, neurotoxicity accounts for approximately 11% of all complications in children treated for acute lymphoblastic leukemia (ALL), encompassing conditions such as peripheral neuropathy, myelosuppression, seizures, aphasia, cognitive impairment, leukoencephalopathy, paresthesia, headaches, and strokes [19]. A 2006 study by Santoro et al. included 2318 children with ALL treated across 43 centers following the AEIOP-BFM protocol. The study used questionnaires to assess the incidence of strokes, which occurred in 11 patients (0.47%). The clinical presentation varied by age: younger children showed irritability, decreased consciousness, and seizures, while older patients reported headaches, hemiparesis, visual and speech disturbances, cranial nerve palsy, ataxia, and seizures. Genetic testing revealed the *TT677 MTHFR* polymorphism in 3 cases; no patients had factor V Leiden or prothrombin gene mutations. In 7 of the 11 cases, antithrombotic treatment—such as unfractionated heparin, low-molecular-weight heparin, or aspirin—was administered, while 4 received no anticoagulation [20]. Also in 2006, the Childhood Cancer Survivor Study group published a retrospective analysis of patients treated for leukemia or brain tumors who experienced a stroke within five years post-treatment. A total of 37 leukemia survivors and 63 brain tumor survivors reported late-onset strokes, with a significantly increased risk observed in those who had received radiation therapy at doses exceeding 30 Gy [21]. In 2013, a study was published analyzing a cohort of children treated for cancer between 2000 and 2009 at a single clinic in the United States. The group included 1411 patients, 15 of whom were diagnosed with strokes. These cases consisted of 7 intracerebral hemorrhages, 5 ischemic strokes, and 3 venous thromboses. Strokes occurred, on average, five months after the cancer diagnosis. Thirteen children died, including six of the seven with intracerebral hemorrhages, who died within seven days of stroke onset [22]. In a retrospective study conducted in the United Kingdom, cases of cerebral venous sinus thrombosis (CVST) were assessed among 3126 participants. CVST was identified in 45 patients, with a mean age of 11 years. More than half of the affected individuals belonged to the high-risk group. All cases were symptomatic, with hemiparesis occurring most frequently (65%), followed by seizures and headaches. Four patients from this cohort died due to complications related to CVST [23]. Another reported case involved a 13-year-old male from China undergoing treatment for acute lymphoblastic leukemia (ALL), who developed an ischemic stroke 30 days after receiving chemotherapy including steroids and asparaginase. He presented a severe headache along with seizures. MR venography revealed superior sagittal sinus thrombosis with hemorrhagic changes in the bilateral frontoparietal lobes. Management included anticonvulsants, nadroparin, and the replacement of pegylated asparaginase with Erwinia-derived asparaginase. Although the patient recovered without neurological deficits, the complication led to a modification of therapy and a delay in intrathecal treatment [24].

Given the increased risk of mortality and thromboembolic events associated with acute lymphoblastic leukemia treatment, the assessment of prothrombotic factors is a key component in optimizing thromboembolic risk management in pediatric patients. A 2016 publication by Boersma et al. measured factor VIII (FVIII), plasminogen activator inhibitor, protein C, and free protein S levels in patients undergoing oncological treatment for hematological malignancies. Elevated factor VIII levels were observed in those who developed thrombosis, likely reflecting the acute-phase reactivity of this procoagulant factor [25]. In a 2009 study by Vormittag et al. involving 840 patients, high factor VIII concentrations were identified as a significant risk factor for symptomatic venous thromboembolism in cancer patients. The results clearly showed that the thrombotic risk associated with factor VIII rose progressively, with even small increases having a substantial impact. Specifically, for every 20% increase in FVIII levels, the risk of thromboembolic events rose by up to 120%. Further analyses indicated that elevated FVIII levels were particularly associated with a higher risk in younger patients [26]. In another study published in 2019, 171 patients undergoing oncological treatment were evaluated, of whom 12 developed thromboembolism. Biomarkers such as D-dimers, fibrinogen, antithrombin, von Willebrand factor, tumor necrosis factor, and IL-6 were assessed in all participants. Among these, von Willebrand factor and IL-6—markers of coagulation and inflammation—showed a significant association with patient mortality [27]. Such biomarker-based insights open opportunities for tailoring thromboprophylaxis to the individual coagulation profile of each patient, potentially improving both safety and efficacy.

Given their potentially severe consequences, thromboembolic events require prompt and effective prevention as well as appropriate treatment strategies. Low-molecular-weight heparin (LMWH) is the preferred drug, owing to its favorable pharmacological properties, including minimal drug interactions and the ability to reverse its anticoagulant effect by withholding the medication for at least 24 h. Other anticoagulants being considered include oral anticoagulants and antithrombin III [28]. A multicenter study conducted in the Netherlands involving children aged 1 to 19 with a first diagnosis of acute lymphoblastic leukemia was registered in 2014. The children were treated according to the Dutch Childhood Oncology Group (DCOG) ALL-11 or ALL-12 protocols. A total of 324 patients were enrolled and divided into two groups. Arm A consisted of patients receiving low-molecular-weight heparin as thromboprophylaxis during the PEG-asparaginase protocol, while Arm B, the standard group, included patients who did not receive prophylaxis. The primary endpoint was symptomatic thrombosis, with secondary endpoints including overall survival and both symptomatic and asymptomatic thrombosis. All patients in Arm A received low-molecular-weight heparin subcutaneously at a dose of 85 IU/kg body weight across all treatment cycles, starting on the day of PEG-asparaginase administration and continuing until day 28 (or day 7 for Erwinase) after the completion of the drug. The results from this study are still pending [29]. Another study investigating the efficacy of anticoagulants was the PARKAA trial, which focused on antithrombin III (AT-III) replacement in children with acute lymphoblastic leukemia undergoing asparaginase therapy. The trial included 85 children, with the treatment arm receiving AT-III replacement when levels fell below 30 U/L. TE occurred in 7 of 25 patients treated with AT-III, compared with 22 of 60 patients whose AT-III levels were not corrected [30]. The THROMBOTECT study was a large prospective randomized trial involving 949 children with newly diagnosed ALL. Participants were randomly assigned to one of the following three groups: those receiving unfractionated heparin, prophylactic low-molecular-weight heparin (LMWH), or antithrombin III (AT-III) replacement therapy. Thromboembolic events occurred in 42 patients (4.4%). Those assigned to receive unfractionated heparin had a higher incidence of TE (8.0%) compared with those treated with LMWH (3.5%; *p* = 0.011) or AT-III (1.9%; *p* < 0.001). The 5-year event-free survival (EFS) was 80.7 ± 2.2% for patients in the AT-III arm, compared with 85.9 ± 2.0% in the enoxaparin group (*p* = 0.10). Both prophylactic AT-III and enoxaparin significantly reduced the incidence of thromboembolic events [31].

The analyzed cases highlight the following three key issues: an increased risk of thromboembolic disease in pediatric cancer patients, the need for universally applicable markers to guide effective prophylaxis, and the lack of clear, standardized guidelines for antithrombotic use in pediatric oncology. From our experience, monitoring the initial clotting times, fibrinogen, and antithrombin III levels as well as factor VIII, von Willebrand factor activity and von Willebrand antigen is an advisable action to detect an increased risk of thrombosis. In the case of elevated levels of prothrombotic factors, it seems safe to use prophylaxis with low-molecular-weight heparin administered subcutaneously. If thrombosis is suspected, we recommend an immediate assessment of the coagulation system and imaging tests. Platelet monitoring is required when using low-molecular-weight heparin. The platelet count with subcutaneous heparin administration should be at least 50,000/μL. In exceptional cases, when it is impossible to administer heparin subcutaneously, an intravenous injection may be chosen.

## 5. Conclusions

Thromboembolic events represent a significant and potentially serious complication in pediatric patients undergoing treatment for acute lymphoblastic leukemia. Their multifactorial etiology—including the malignancy itself, chemotherapeutic agents, and supportive care measures such as central venous catheters—requires special clinical attention. Among the possible complications, neurological manifestations can occur and should not be overlooked in the overall risk assessment. The early identification and management of thrombotic risk are essential to minimize both acute and long-term consequences. Despite growing awareness and numerous clinical studies, standardized prophylactic guidelines are lacking, resulting in varying practices across treatment centers. Evidence supports the utility of monitoring specific coagulation parameters—such as factor VIII, von Willebrand factor, fibrinogen, and antithrombin III—to help identify patients at increased risk. In such cases, the prophylactic administration of low-molecular-weight heparin (LMWH) may serve as a safe and effective strategy. At our center, this approach is routinely applied to every patient with an oncology diagnosis. To date, we have not observed any serious side effects associated with the use of low-molecular-weight heparin (LMWH). This individualized, factor-driven approach reflects the principles of personalized medicine, allowing for prophylaxis tailored to the patient’s specific risk profile and laboratory parameters. By moving beyond generalized risk models and integrating patient-specific data into decision-making, we contribute to a more precise and patient-centered strategy for thromboembolic prevention in pediatric oncology. Nevertheless, the establishment of unified, evidence-based protocols remains crucial for improving outcomes and ensuring consistent care for pediatric oncology patients across all treatment facilities.

## Figures and Tables

**Table 1:** Admission laboratory test results.

Parameter	Results
WBC * (×10^3^/uL)	**171,370**
Neutrophils (×10^3^/uL)	**1050**
Lymphocytes (×10^3^/uL)	**137,660**
Monocytes (×10^3^/uL))	**32,500**
Blasts (%)	**89**
Hg (g/L)	**7.4**
PLT (×10^3^/uL)	**20,000**
AspAT(U/L)	14
AlAT (U/L)	14
LDH (U/L)	**471**
CRP (mg/dL)	**1.39**
Uric acid (mmol/L)	0.20
Creatinine (mg/dL)	0.77
Urea (mg/dL)	34.9
IgG (mg/dL)	1155
PT (s)	14.3
INR	1.21
APTT (s)	30.3
TT (s)	17.3
AT-III (%)	96
Fibrinogen (g/L)	2.91
Protein C (%)	67
Protein S (%)	**41.2**
Factor V Leiden	3.05
Factor VIII (%)	109
von Willebrand factor—activity (%)	116
von Willebrand factor—antigen (%)	**180**

* WBC—white blood cell; Hg—hemoglobin; PLT—platelet; AspAT—aspartate aminotransferase; AlAT—alanine aminotransferase; LDH—lactate dehydrogenase; CRP—C-reactive protein; PT—prothrombin time; INR—International Normalized Ratio of Prothrombin; APTT—partial thromboplastin time; TT—thrombin time; AT-III—antithrombin III. Abnormal results are in bold.

**Table 2:** Characteristics of the currently used protocol.

Course of the Protocol	Drugs
Protocol I	Prednisone, vincristine, daunorubicin, and *Escherichia coli* PEG-asparaginase,
Consolidation A and B	Cytarabine, 6-mercaptopurine, and cyclophosphamide
Protocol M	6-Mercaptopurine and high-dose methotrexate
Protocol II	Dexamethasone, vincristine, doxorubicin, *Escherichia coli* PEG-asparaginase, cyclophosphamide, cytarabine, and 6-thioguanine
Maintenance Therapy	6-Mercaptopurine and methotrexate

**Table 3:** The dynamics of selected factors during Protocol II.

	Day 0	Day 21	Day 28	Day 35	Day 49	Day of the Stroke
Factor VIII (%)	143	**301**	**323**	**200**	74	103
von Willebrand factor—activity (%)	235	**508**	**511**	**319**	112	159
von Willebrand factor—antigen (%)	171	**757**	**769**	**183**	157	172
AT-III * (%)	120	63	83	86	105	85%
Fibrinogen (g/L)	2.47	0.55	0.49	3.46	**5.01**	**6.25**

* AT-III—antithrombin III. Test results above the norm are in bold, and below are underlined.

**Table 4:** Laboratory test results performed on the day of the stroke.

Parameter	Results
WBC * (×10^3^/uL)	**0.73**
Neutrophils (×10^3^/uL)	**0.59**
Lymphocytes (×10^3^/uL)	**0.13**
Monocytes (×10^3^/uL)	**0.01**
Hg (g/L)	**9.2**
Ht (%)	**25.7**
Reticulocytes (‰)	**1.9**
PLT (×10^3^/uL)	**22,000**
AspAT(U/L)	**51**
AlAT (U/L)	18
LDH (U/L)	181
CRP (mg/dL)	**1.66**
Creatinine (mg/dL)	0.30
Urea (mg/dL)	26
IgG (mg/dL)	813
Total cholesterol (mg/dL)	117
Cholesterol LDL (mg/dL)	68
Cholesterol HDL (mg/dL)	35
Cholesterol non-HDL (mg/dL)	82
Triglycerides (mg/dL)	69
PT (s)	12.5
INR	1.06
APTT (s)	32.4
AT-III (%)	85
Fibrinogen (g/L)	**6.25**
D-dimers (ng/mL)	**536**
Protein C (%)	72
Protein S (%)	90.5
Factor V Leiden	3.02
Factor VIII (%)	103
von Willebrand factor—activity (%)	159
von Willebrand factor—antigen (%)	172
Lupus anticoagulant (total ratio)	1.10
Beta-2 glycoprotein antibody IgM	<2.00
Beta-2 glycoprotein antibody IgG	<2.00
Anti-cardiolipin antibodies IgM	<2.00
Anti-cardiolipin antibodies IgG	<2.00

* WBC—white blood cell; Hg—hemoglobin; Ht—hematocrit; PLT—platelet; AspAT—aspartate aminotransferase; AlAT—alanine aminotransferase; LDH—lactate dehydrogenase; CRP—C-reactive protein; LDL—low-density lipoprotein; HDL—high-density lipoprotein; non-HDL—non-high-density lipoprotein; PT—prothrombin time; INR—International Normalized Ratio of Prothrombin; APTT—partial thromboplastin time; TT—thrombin time; AT-III—antithrombin III; Abnormal results are in bold.

## Data Availability

No new data were created or analyzed in this study. Data sharing is not applicable to this article.

## Abbreviations

The following abbreviations are used in this manuscript:

AIEOP-BFM	International Collaborative Treatment Protocol for Children and Adolescents with Acute Lymphoblastic Leukemia Berlin-Frankfurt-Muenster
AlAT	Alanine aminotransferase
ALL	Acute lymphoblastic leukemia
APTT	Partial thromboplastin time
ASP	Asparaginase
AspAT	Aspartate aminotransferase
AT-III	Antithrombin III
CNS	Central nervous system
CRP	C-reactive protein
CSVT	Cerebral venous sinus thrombosis
CVC	Central venous catheter
CYP-C	Cancer in Young People Canada
DCOG	The Dutch Childhood Oncology Group
EFS	Event-free survival
F2	Coagulation Factor II
F5	Coagulation Factor V
HDL	High-density lipoprotein
Hg	Hemoglobin
Ht	Hematocrit
INR	International Normalized Ratio of Prothrombin
LDH	Lactate dehydrogenase
LDL	Low-density lipoprotein
LMWH	Low-molecular-weight heparin
NHL	Non-Hodgkin lymphoma
non-HDL	Non-high-density lipoprotein
PLT	Platelet
PT	Prothrombin time
TE	Thromboembolism
TT	Thrombin time
VTE	Venous thromboembolism
vWF	von Willebrand factor
WBC	White blood cell
